# Implications for conservation assessment from flux in the botanical record over 20 years in southwest Ghana

**DOI:** 10.1002/ece3.9775

**Published:** 2023-01-24

**Authors:** Cicely A. M. Marshall, Jonathan Dabo, Markfred Mensah, Patrick Ekpe, William D. Hawthorne

**Affiliations:** ^1^ Department of Plant Sciences University of Cambridge Cambridge UK; ^2^ Forestry Research Institute of Ghana Kumasi Ghana; ^3^ Ghana Herbarium, Department of Plant & Environmental Biology University of Ghana Legon Ghana; ^4^ Department of Plant Sciences University of Oxford Oxford UK

**Keywords:** conservation, endemism, global plant inventory, species discovery, taxonomy, tropical biodiversity

## Abstract

At best, conservation decisions can only be made using the data available at the time. For plants and especially in the tropics, natural history collections remain the best available baseline information upon which to base conservation assessments, in spite of well‐documented limitations in their taxonomic, geographic, and temporal coverage. We explore the extent to which changes to the plant biological record over 20 years have changed our conception of the conservation importance of 931 plant taxa, and 114 vegetation samples, recorded in forest reserves of the southwest Ghana biodiversity hotspot. 36% of species‐level assessments changed as a result of new distribution data. 12% of species accepted in 2016 had no assessment in 1996: of those, 20% are new species publications, 60% are new records for SW Ghana, and 20% are taxonomic resolutions. Apparent species ranges have increased over time as new records are made, but new species publications are overwhelmingly of globally rare species, keeping the balance of perceived rarity in the flora constant over 20 years. Thus, in spite of considerable flux at the species record level, range size rarity scores calculated for 114 vegetation samples of the reserves in 1996 and 2016 are highly correlated with each other: *r*(112) = 0.84, *p* < .0005, and showed no difference in mean score over 20 years: paired *t*(113) = −0.482, *p* = .631. This consistency in results at the area level allows for worthwhile conservation priority setting over time, and we argue is the better course of action than taking no action at all.

## Graphical abstract

At best, conservation decisions can only be made using the data available at the time. We explore the extent to which changes to the plant biological record over 20 years have changed our conception of the conservation importance of 931 plant taxa, and 114 vegetation samples, recorded in forest reserves of the south west Ghana biodiversity hotspot. 36% of species‐level assessments changed as a result of new distribution data, and 12% of currently accepted species have been added to the checklist since 1996. In spite of considerable flux at the species record level, range size rarity scores for vegetation samples have stayed remarkably constant over 20 years. This consistency in results at the area level allows for worthwhile conservation priority setting over time.

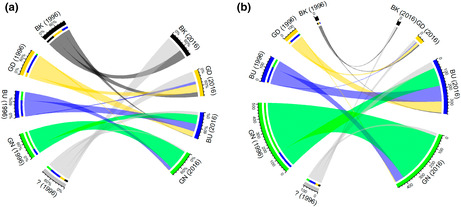

## 1 INTRODUCTION

At best, conservation decisions can only be made using the data available at the time. Short of surveying a whole region to make decisions locally, the best we can hope to do is to use all available data and to characterize how our current data may be biasing our perspective. For plants, the most commonly used baseline information upon which conservation assessments are based is the geographic distribution of species in terms of area of occupancy (AOO) or extent of occupancy (EOO) as revealed by herbarium specimens (Brummitt, Bachman, Griffiths‐Lee, et al., 2015). While there have been significant efforts to digitize and curate herbarium records (Dauby et al., 2016; Enquist et al., 2016), the expense of collecting and storing specimens long‐term has limited the plant biological record in terms of its taxonomic, geographic, and temporal coverage and resolution (Meyer et al., 2016; Roberts et al., 2016; Schmidt‐Lebuhn et al., 2013; Stropp et al., 2016). The principle problem with an incomplete baseline description of plant species' distributions for conservation is that newly accessioned specimens (or new field observations) will tend to produce increases in EOO and AOO (Brummitt, Bachman, Aletrari, et al., 2015).

If our evidence threshold for conservation assessment is too stringent, we fail to define a baseline against which to monitor change for many species. If our evidence threshold is set too low, we risk producing misleading or labile conservation assessments inappropriate for a legal framework. Protecting “undeserving” species has economic costs, while failing to protect “deserving” species results in local or global extinctions. At the moment, our evidence threshold for conservation assessment via the IUCN Red List is set very high for plants. Authors need “good evidence to establish that a species is not undercollected” in order to publish an IUCN Red List assessment for a species with <5 records (Brummitt, Bachman, Aletrari, et al., 2015), while 15 georeferenced specimens per species are needed to produce Red List range estimates consistent with estimates based on all known specimens (Rivers et al., 2011). Fifteen distinct georeferenced specimens are a high bar for most plant species. It takes around 70 years from the collection of the first specimen to reach 15 specimens (Goodwin et al., 2020). 36.5% of the world's plant species (158,535 species) are represented by five herbarium or field observations or fewer, with 28.3% (123,149 species) having three observations or fewer, and 13.6% to 11.2% species with just one observation (Enquist et al., 2019). The result of this high burden of evidence is that Red Listing progress for plants has been limited, at around 8.5% of accepted vascular plant species names, excluding those in need of updating (26,720 of 316,143). As much as 39% of plant species selected randomly for inclusion in the Sampled Red List Index for Plants could not be evaluated against the Red List criteria, even as Data Deficient (Brummitt, Bachman, Griffiths‐Lee, et al., 2015). For the 5 years preceding 2019, substantially fewer species were assessed for the Red List each year than were published the same year, resulting in negative progress in real terms after the historical synonymy rate of 0.34% is applied.

In Ghana, the Star system has been used since 1996 to prioritize plant species for conservation (Hawthorne, 1996). In the Star system, species are assigned to one of four categories of global range size rarity by reference to their area of occupancy (AOO) measured globally with a fixed cell size of 1 degree square (or 100 × 100 km outside the tropics). Black Star species are the most globally rare species, with a mean global AOO of 2.7 degree squares. Gold Star species have restricted ranges of 8 degree squares on average, Blue Star species have a three times larger range of 24 degree squares, while Green Star species are the most widespread. One of the tenets of Star rating is that all species should be rated using the best information available at the time, even if this is perhaps incomplete, with ratings being updated in light of new information if necessary (Hawthorne & Marshall, 2016). There is also no requirement to show a decline in population size or distribution, as there is for the Red List, which reduces the burden of proof further. All plant species in tropical Africa have a published Star rating (Marshall et al., 2016). Black Star species are legally protected from logging in Ghana: The Ghanaian Government's Timber Resources Management Act (Act 547) and Timber Resources Management Regulations (LI 1649) of 1998 underpin current forest regulations, and the enforced logging manual is framed in the context of Star categories, where Black Star species are wholly protected from logging.

In Ghana, among other countries, area‐based conservation action has also been taken on the basis of the Star ratings. Each Star category carries a weight in inverse proportion to its rarity. The weights of the species present in a vegetation sample are averaged to give a *bioquality* score for the vegetation overall, reflecting the concentration of globally rare plants within the vegetation. In 1999, the Ghanaian Government established the High Forest Biodiversity Conservation Project (HFBCP) funded by the World Bank, which aimed to conserve biodiversity in these forests through the establishment and protection of Globally Significant Biodiversity Areas (GSBAs). These reserves were identified and protected on the basis of their high bioquality scores. This approach led to the establishment of 29 forest reserves amounting to c. 2300 km^2^ of forest reserves or 13% of the total forest network and their exclusion from timber harvesting. In 2009, Ghana was the first country to sign a Forest Law Enforcement Governance and Trade (FLEGT) Voluntary Partnership Agreement (VPA) with the EU (The European Community and the Republic of Ghana, 2010). As a condition of the VPA, it is stated that no Black Star species, as defined in Hawthorne, 1996, can be felled and that no timber can come from a GSBA. By 2007, the GSBAs showed improved afforestation and rainfall patterns, reduced illegal tree felling and group hunting, less seasonal reduction of volumes of water bodies, and the cessation of the use of poisonous chemicals in fishing (GEF Evaluation Office–UNDP Evaluation Office, 2007).

Hotspots of locally endemic or restricted range species are treated as priority areas for plant conservation well beyond Ghana's GSBAs, for example, recently in the Key Biodiversity Areas designation (Brooks et al., 2006; IUCN, 2016a; Myers et al., 2000). There has been some work addressing how uncertainty in our knowledge of plant species' taxonomy and distribution manifests in lability of species' IUCN ratings over time (Goodwin et al., 2020; Lughadha et al., 2019; Rivers et al., 2011). However, how incomplete knowledge at the species level manifests in conservation assessments of areas is an important and underexplored question (Daru et al., 2020; Murray‐Smith et al., 2009; Nelson et al., 1990).

Here, we describe changes in the nomenclature and documented distributions of the vascular plant species of the southwest Ghana forest reserves over 20 years (1996 to 2016). We explore the consequence of these changes for species‐level conservation assessments (Star ratings) and area‐based conservation assessments (bioquality scores). We argue that in spite of considerable flux in the botanical record, area‐based conservation assessments in particular have remained remarkably stable over 20 years and have led to positive outcomes for conservation in Ghana. By contrast, forests outside of reserves experienced significant and growing threats from logging, fuelwood collection, agriculture, mining, and climate change (Aleman et al., 2018; Edwards et al., 2014; McClean et al., 2006).

## 2 MATERIALS AND METHODS

### 2.1 Study area

Upper Guinea is a phytogeographical name for the forest zone of west Africa, running from Sierra Leone in the west to the Dahomey Gap (Ghana) in the east, from the coast up to 350 km inland (Marshall et al., 2021; White, 1979). This area is included within the Western African Forests ecoregion, which has been recognized as a biodiversity hotspot (Myers et al., 2000; Olson et al., 2001). The forest reserves of SW Ghana, where this study is situated, have also been recognized as Key Biodiversity Areas (KBAs) (Key Biodiversity Areas Partnership, 2022). Southwest Ghana is regarded as a hotspot of plant endemism within west Africa, being home to a high concentration of globally rare species (Bongers et al., 2004; Marshall et al., 2016, 2022). The typical flora of the study area is lowland evergreen rainforest, with variations in species composition driven by gradients in rainfall, disturbance, and local topology, with altitude and historical climatic stability important at broader geographical scales (Marshall et al., 2022). Across the sampled sites, annual precipitation averages 1910 mm/year, while mean temperatures average 26.5°C (Hijmans et al., 2005). Altitude above sea level has a minimum of 40 m, a maximum of 181 m, and a mean of 97 m. The forest reserves of southwest Ghana are subject to anthropogenic disturbance, for example, accommodating logging concessions.

### 2.2 Field methods

**FIGURE 1:**
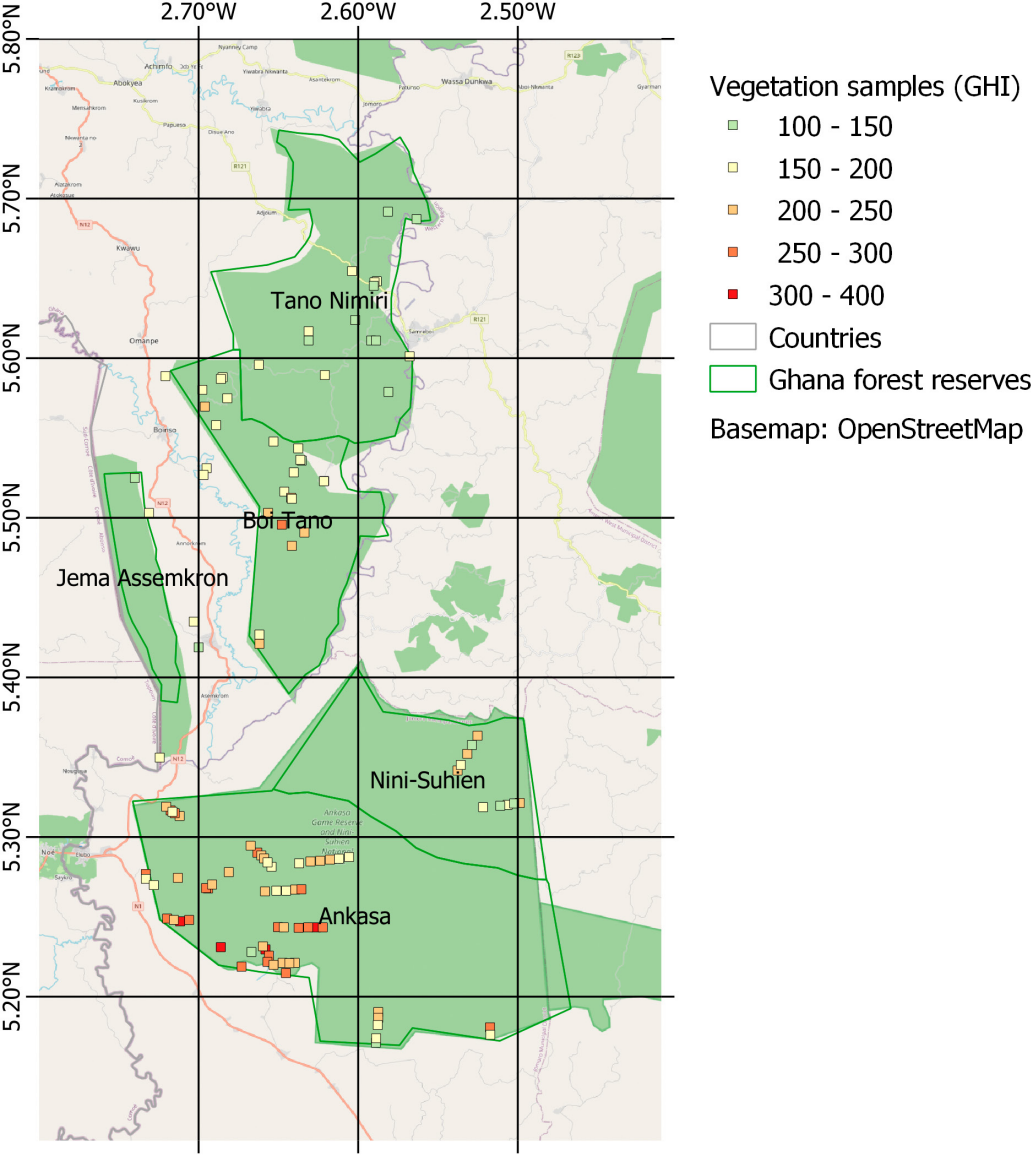
Map of 114 sample locations in the southwest Ghana study area, colored by GHI calculated using the Stars published in 2016 and including all species found. Reserves are named.

Twenty‐seven samples were enumerated in 2015 for this publication and were compiled with 87 botanic samples from the region from different surveys (Table 1). The combined dataset contains 12,232 records of 931 taxa identified to species level, from 114 samples. With the exception of the Hall & Swaine, 1981 A plots, which were 25 × 25 m samples of the vascular flora, and B plots (the first 30–60 vascular plant species encountered; Hall & Swaine, 1981), all samples were conducted using the Rapid Botanic Survey (RBS) method (Hawthorne & Marshall, 2016). RBS samples were bounded by survey effort rather than size, with the aim of recording all vascular plant species within a specified vegetation type and landscape unit, and enumerating at least 40 individual canopy trees. Identification and fieldwork was carried out by JD, MM, CM, and WH, with assistance at the University of Ghana herbarium (GC) from PE. Permission to collect and export plants was obtained from the Ministry of Lands and Natural Resources, Samartex, and the Wildlife Division for Ankasa RR. Specimens were collected of all plants for which identification was not absolutely certain; these are housed at the Daubeny herbarium, Oxford. The 114 sample locations (Figure 1) were situated in order to capture variation in forest type, condition, and geography across the five forest reserves, as all the surveys had as their primary goal the purpose of baselining or inventorying the forest reserves. Mapping was conducted in QGIS version 2.16.1.

**TABLE 1:** Datasets compiled for SW Ghana

Dataset	Reference/ownership	No. of samples: Ankasa	Boi Tano	Tano Nimri	Jema Assemkron	Nini Suhien	Total
2015RBS	Marshall et al. (this publication)	2	16	9	0	0	27
2015TSP	Marshall et al. (this publication)	3	0	0	0	0	3
HANDS	Hall and Swaine (1981)	2	0	2	1	0	5
RBS1991	Hawthorne and Abu‐Juam (1995), Hawthorne (1996). Ghana Forestry Dept.	7	4	4	3	0	18
ANK1998	Hawthorne (1998). Ghana Wildlife Dept.	45	0	0	0	10	55
GSBA	Hawthorne (2002)/Ghana Biodiversity Unit Ministry of Lands & Forestry	0	5	0	1	0	6
		59	25	15	5	10	114

### 2.3 Conservation ratings over time

#### 2.3.1 Species

We use Star ratings as our species‐level conservation assessment (Hawthorne & Marshall, 2016; Marshall et al., 2016). Species are assigned to one of four categories of range size rarity, measured by reference to their area of occupancy (AOO) measured globally with a fixed cell size of 1 degree square (or 100 × 100 km outside the tropics). Black Star species have a mean global AOO of 2.7 degree squares, Gold Star of 8, Blue of 24, and Green of 72. For species or regions with poor coverage in terms of published degree square location records, proxies such as districts or prefecture‐level occurrence data have been used for assigning Stars. One degree (or 100 km) square is an appropriate grid size for analyzing plant species' ranges at global or continental scope, reflecting the (lack of) spatial resolution available in the herbarium record for most plant species worldwide.

Star ratings for 1403 species of the forest zone of Ghana were published in 1996, using the best available distribution data available at the time (Hawthorne, 1996). The basis of this was the range descriptions in the Flora of West Tropical Africa (FWTA second edition, 1952–1972), along with field and herbarium records collected by or shared with the last author (WH). In 2016, Star ratings for c. 40,000 African plant taxa were published from an updated curated database describing the global range of tropical African plant species (Marshall et al., 2016). The method of assessment was the same, while the available data were different.

We examined the congruence between these 1996 and 2016 Star ratings, for 931 currently accepted plant species recorded at least once in 114 vegetation plots in southwest Ghana. All species distribution records from 114 vegetation samples (Table 1) were collected together in a single database table (see ‘Section 2.2’ for vegetation sampling detail). All records were analyzed at species level, with subspecies and varieties reduced to species level, and any record identified to genus level or higher only also removed. All records were converted to their currently accepted name (current as of 2016), following the African Plants Database (Conservatoire et Jardin botaniques de la Ville de Genève and South African National Biodiversity Institute Pretoria, 2016). Two hundred and fifty species names in the original records are now considered synonyms. Nine of those records were then redundant, as they had been sunk into names already listed (1 Black, 2 Gold, 2 Blue, and 4 Green). Homotypic synonyms with a merely cosmetic name change and no associated range change are treated for this analysis as if they remained unchanged, that is, they are not included in the counts of changes or additions. Reddish Stars (S, R, P) and savanna (X) Stars published in 1996 were excluded from the dataset and calculations, as these are no longer in use.

A species' Star rating may *change* for two reasons: (i) new distribution data alters our understanding of the species' range; (ii) the species experiences a genuine increase or decrease in range size. Star ratings may be *added* when (1) new species are published (and found in southwest Ghana), including the splitting of taxa; (2) previously published species are newly recorded in (southwest) Ghana; (3) “known unknowns” are resolved, that is, species which were considered to be of uncertain Star in 1996 are resolved to a Star in 2016 thanks to new sources of information. This flux is visualized with a chord diagram implemented with R package circlize (Gu et al., 2014; R Core Team, 2020).

#### 2.3.2 Areas

We use the Genetic Heat Index (GHI) as our area‐level conservation rating (Hawthorne, 1996; Hawthorne & Marshall, 2016), the standard index of bioquality published in 1996 and used to designate Ghana's GSBAs. GHI is a continuous metric representing the weighted global ranges (endemism) of the species present in a plant community. The calculation is similar to range size rarity, except that species' ranges are measured globally at one degree square resolution rather than within the study area at any resolution, species ranges are categorized into four groups, called Stars, rather than being treated continuously, and bioquality includes no measure of species richness, as is sometimes the case with range size rarity metrics.

GHI is calculated as the proportion of species belonging to each Star rating within a sample, where each species is inversely weighted by the mean range size of its Star (BK = 27, GD = 9, BU = 3, GN = 0). The GHI for each sample was calculated from the species present, using the following formula, where NBK, NGD, NBU, and NGN are the number of Black, Gold, Blue, and Green Star species in a sample, and WBK, WGD, and WBU are the respective weights.

$$\mathrm{GHI}=100\times \left(\left(\mathrm{NBK}\times \mathrm{WBK}\right)+\left(\mathrm{NGD}\times \mathrm{WGD}\right)+\left(\mathrm{NBU}\times \mathrm{WBU}\right)+\left(\mathrm{NGN}\times \mathrm{WGN}\right)\right)/\left(\mathrm{NBK}+\mathrm{NGD}+\mathrm{NBU}+\mathrm{NGN}\right)$$

To investigate the change in these area‐level conservation assessments over time, GHI was calculated for 114 vegetation samples using (i) the Stars published in 1996, and (ii) the Stars published in 2016. Records identified to genus or family level only were dropped from the datasets. Analysis was conducted at the lowest named taxonomic level (species or named infraspecific taxa). Any record carrying a synonymous name was updated to the accepted name before analysis, using the taxonomic framework of the African Plants Database. GHIs calculated as they appeared in 1996, and 2016, are compared with each other via paired t‐test, Pearson correlation, and linear regression in base R (R Core Team, 2020).

## 3 RESULTS

### 3.1 Stability of species‐level conservation assessments

**FIGURE 2:**
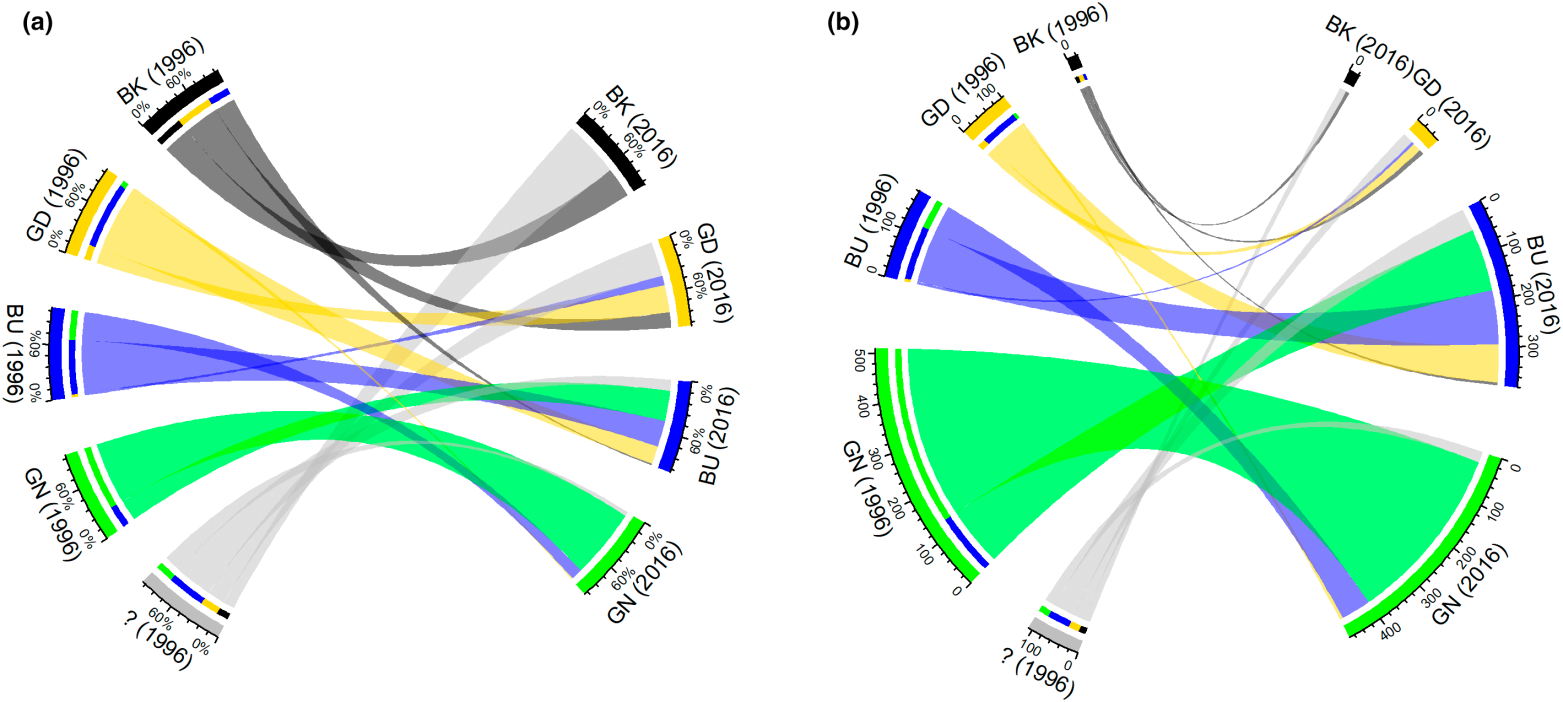
Chord diagrams showing the conservation assessment trajectory of 931 currently accepted Ghanaian forest zone species recorded at least once in the reserves. (a) Chord and segment widths are proportional to the number of species in each category. (b) segment widths are scaled equally.

Changes and additions to published Star ratings over 20 years are explored for the forest zone of southwest Ghana (Table 2). All species recorded at least once in the reserves are considered, including species which were not refound in 2015 and species which did not have a Star rating in 1996. Of 931 currently accepted species found in the 114 samples of the forest reserves of Ghana, half (51%) are currently considered Green Star (the most widespread species) and 41% are Blue Star (the second most widespread rating). Just 2.5% of species are considered Black Star (the most range‐restricted category), and 6% are Gold Star (Table 2). There has been considerable flux in the identity of the species present in each of the Star ratings (Figure 2).

**TABLE 2:** Star ratings for all 931 currently accepted species recorded at least once in the 114 samples of the reserves of southwest Ghana.

Star	BK (2016)	GD (2016)	BU (2016)	GN (2016)	
BK (1996)	8	10	6	0	24
GD (1996)	0	18	80	7	105
BU (1996)	0	6	114	63	183
GN (1996)	0	0	132	378	510
Unknown (1996)	15	23	48	23	109
	23	57	380	471	931

*Note*: Star ratings in 1996 are compared with their destination rating in 2016. Column totals show the total number of species in each Star rating in 2016; row totals show the total number of species with each Star rating in 1996. To avoid double counting, species from 1996 which have since been synonymized to species already included are not repeated, so 24 rather than 25 BK star species of 1996 are referred to here (one Black Star species *Trichoscypha chevalieri* was sunk to *T. lucens*, already a Green Star species in 1996).

#### 3.1.1 Changes

Considering only the species which had a known Star rating in 1996 (species with no Star rating in 1996 are excluded), 36% of 1996 ratings are different now, while 64% of 1996 assessments have remained stable (Table 3). Of the ratings which changed, a slightly higher proportion of species were downgraded compared with upgraded: 20% of species ratings were downgraded, compared with 16% of species ratings upgraded.

**TABLE 3:** Changes to the Star ratings published in 1996. The proportion of 1996 Star ratings which have remained stable (Star rating the same in 2016), have been upgraded (moved to a higher Star rating in 2016), or downgraded (to a lower Star category in 2016), grouped by original Star rating. Species with no Star rating in 1996 are excluded.

	BK (1996)	GD (1996)	BU (1996)	GN (1996)	Overall
Stable	33.3%	17.1%	62.3%	74.1%	64%
Upgraded	–	0%	3.28%	25.9%	16%
Downgraded	66.6%	82.9%	34.4%	–	20%

Commoner species have more stable Star ratings than rarer species: 74% of Green Star species have remained Green Star, compared with 33% of Black Star species which have remained Black Star. For the rarest (Black Star) species, only one third of species considered Black Star in 1996 remain Black Star species in 1996, with the remaining two thirds downgraded to Gold or Blue Star species. A similar pattern of downgrading can be seen for Gold Star species and to a lesser extent Blue Star species.

Downgrading of species from the rare categories to more widespread categories is the result of increases in the documented range of species, due to more distribution data records being collected and becoming available online and for analysis. An inestimable proportion of these records will represent genuine range extensions for species, although the majority are very likely to be simply the result of better recording of established ranges. Overwhelmingly, the upgraded species are species which moved from Green to Blue Star: These are species which appeared to be widespread from their range description in FWTA (e.g., “to Lower Guinea”), but on consideration of their modern dot map distribution data have subsequently been shown to be sparse within that extent of occurrence.

#### 3.1.2 Additions

Of the 931 species now considered to be present in samples of southwest Ghana, 109 species (12%) had no Star rating in 1996 (Table 4).

**TABLE 4:** New additions to the flora of southwest Ghana are overrepresented in the 2016 Black and Gold Star ratings and underrepresented in the 2016 Green Star ratings

	BK (2016)	GD (2016)	BU (2016)	GN (2016)	
Species Star rating additions since 1996	15 (expected 2.7)	23 (expected 6.7)	48 (expected 45)	23 (expected 55)	109
Remainder (changes plus constant)	8	34	332	448	822
	23	57	380	471	931

*Note*: Chi‐square test of association, *X*‐squared = 130.48, df = 3, *p* < .005.

New additions to the flora are disproportionately rare species: A chi‐square test of association shows that many more species additions proved to be Black or Gold Star species than would be expected given previously rated species, and many fewer than expected proved to be Green Star (Table 4). For the rarer Black and Gold Star species, additions contribute a significant proportion of the currently accepted ratings: 65% of Black Star species were unknown in 1996, and 41% of Gold Star species were unknown in 1996.

The majority of additions to the Star‐rated flora were the result of new records for southwest Ghana or Ghana as a whole (60%); 20% are new species publications, and 20% are resolved names. Resolved names are the species whose distributions, or taxonomic status, were uncertain enough in 1996 to be given a Star rating of “?” at the time. Many examples of new species publications (since 1996) are of species currently considered rare, for example, *Pavetta abujuamii* and *P. ankasensis*; *Psychotria hawthornei*, based on new collections; while others were based on reassessment of older specimens and went straight to Blue Star once named, for example, *Eremospatha dransfieldii* and *Hypselodelphys triangularis*.

New records for the region include

1. Species found in southwest Ghana for the first time since 1996, though previously recorded elsewhere in West Africa (e.g., *Tricalysia parva*; *Dichapetalum staudtii*; *Didelotia afzelii*; *Hunteria simii*; *Mussaenda grandiflora*; *Pavetta subglabra*; *Pleioceras afzelii*; and *Whitfieldia colorata*);
2. Species not recorded as present in Ghana by the Flora of West Tropical Africa (FWTA) (e.g., *Chassalia laxiflora* (Liberia only); *Combretum fulvum* (Guinea only); *Crotonogyne caterviflora* (Sierra Leone and Liberia only); and *Crudia klainei* (Ivory Coast, French Cameroons, Gabon));
3. Species not mentioned at all by FWTA, being referred to there as another currently recognized taxon or having been published between FWTA publication and 1996, for example, *Campylospermum laxiflorum*/*Ouratea laxiflora*; *Cissus miegei*; *Cleistanthus ripicola*; *Cnestis bomiensis*, *Geophila flaviflora*; and *Laccosperma acutiflorum*.

#### 3.1.3 Conservation priority species

Changes to the list of species considered to be of the highest conservation priority are detailed in Table 5. Twenty‐three Black star taxa are currently accepted as present in SW Ghana. In 1996, there were 25 Black Star species known from these reserves. Sixteen of those species considered Black Star in 1996 have been downgraded. One species has been sunk to a species already published in 1996 (*Trichoscypha chevalieri* to *T. lucens*, not included in calculations). Fifteen new Black Star species have been added to the list: 10 by new species publications, 4 by new records in the region, and one species considered too uncertain to merit a Star rating in 1996 has been resolved as a Black Star species. Eight Black star species are stable: recorded as Black Star species of the reserves in 1996, and still Black Star species of the reserves in 2016. Stability of area‐based conservation assessments.

**TABLE 5:** Species currently or previously considered Black Star species of southwest Ghana

Species	Star 2016	Star 1996	Notes
*Calycobolus hallianus*	BK		New species
*Chytranthus verecundus*	BK	BK	Stable
*Cola umbratilis*	BK	BK	Stable
*Dorstenia embergeri*	BK	?	Resolved
*Ficus pachyneura*	BK		New record 2015
*Gaertnera luteocarpa* (*subsp. luteocarpa*)	BK		New species
*Hymenostegia gracilipes*	BK	BK	Stable
*Leucomphalos discolor*	BK	BK	Stable
*Momordica sylvatica*	BK		New species
*Monocyclanthus vignei*	BK	BK	Stable
*Pavetta abujuamii*	BK		New species
*Pavetta ankasensis*	BK		New species
*Pavetta sonjae*	BK		New species
*Pavetta subglabra*	BK		New record 1998
*Psychotria ankasensis*	BK	BK	Stable
*Psychotria hawthornei*	BK		New species
*Psychotria longituba*	BK	BK	Stable
*Psychotria nigrostellata*	BK		New species
*Schefflerodendron ekpei*	BK		New species
*Suregada ivorensis*	BK		New record 2015
*Synsepalum ntimii*	BK		New species
*Tapura ivorensis*	BK	BK	Stable
*Tricalysia parva*	BK		New record 2015
*Alsodeiopsis chippii*	GD	BK	Downgraded
*Chrysophyllum azaguieanum*	GD	BK	Downgraded
*Dactyladenia hirsuta*	GD	BK	Downgraded
*Dasylepis blackii*	BU	BK	Downgraded
*Leptoderris cyclocarpa*	GD	BK	Downgraded
*Leptoderris miegei*	GD	BK	Downgraded
*Neolemonniera clit*andrifolia*EN	BU	BK	Downgraded
*Nephthytis swainei*	GD	BK	Downgraded
Pierreodendron kerstingii *VU	GD	BK	Downgraded
Placodiscus bancoensis *VU	GD	BK	Downgraded
*Psychotria brachyanthoides*	BU	BK	Downgraded
*Psychotria subglabra*	GD	BK	Downgraded
*Ruellia togoensis*	BU	BK	Downgraded
*Sclerosperma mannii*	BU	BK	Downgraded
Shirakiopsis aubrevillei *VU	BU	BK	Downgraded
*Synsepalum aubrevillei*	GD	BK	Downgraded
*Trichoscypha chevalieri*	–	BK	Sunk to *T. lucens* (*GN*)

*Note*: Species denoted with asterisk * are species large enough trees to be of potential interest for timber, but are nevertheless still protected from logging as they were designated Black Star in 1996. All are also Red Listed threatened species, EN, Endangered, VU, Vulnerable, and we do not recommend that their protection from logging is removed.

The overall GHI for species of southwest Ghana calculated using the 1996 Stars is 218; the overall GHI for species of southwest Ghana calculated using the 2016 Stars is very similar, at 216. On the whole, the forces of change have converged to give the same overall impression of the balance of rarity and widespreadness in the flora.

This is reflected when we compare the GHIs for each sample calculated using the Stars of 1996, to the GHIs for each sample calculated using the Stars of 2016, via a paired Student's t‐test. GHIs were normally distributed using both 1996 and 2016 Star ratings. The mean difference is negligible at −1.91 GHI points, and the 95% confidence interval (−9.77 to 5.94) includes 0 (paired *t*(113) = −0.482, *p* = .631). Overall, the mean GHI score for the samples is the same, whether scores are calculated with the old Stars or the new Stars.

**FIGURE 3:**
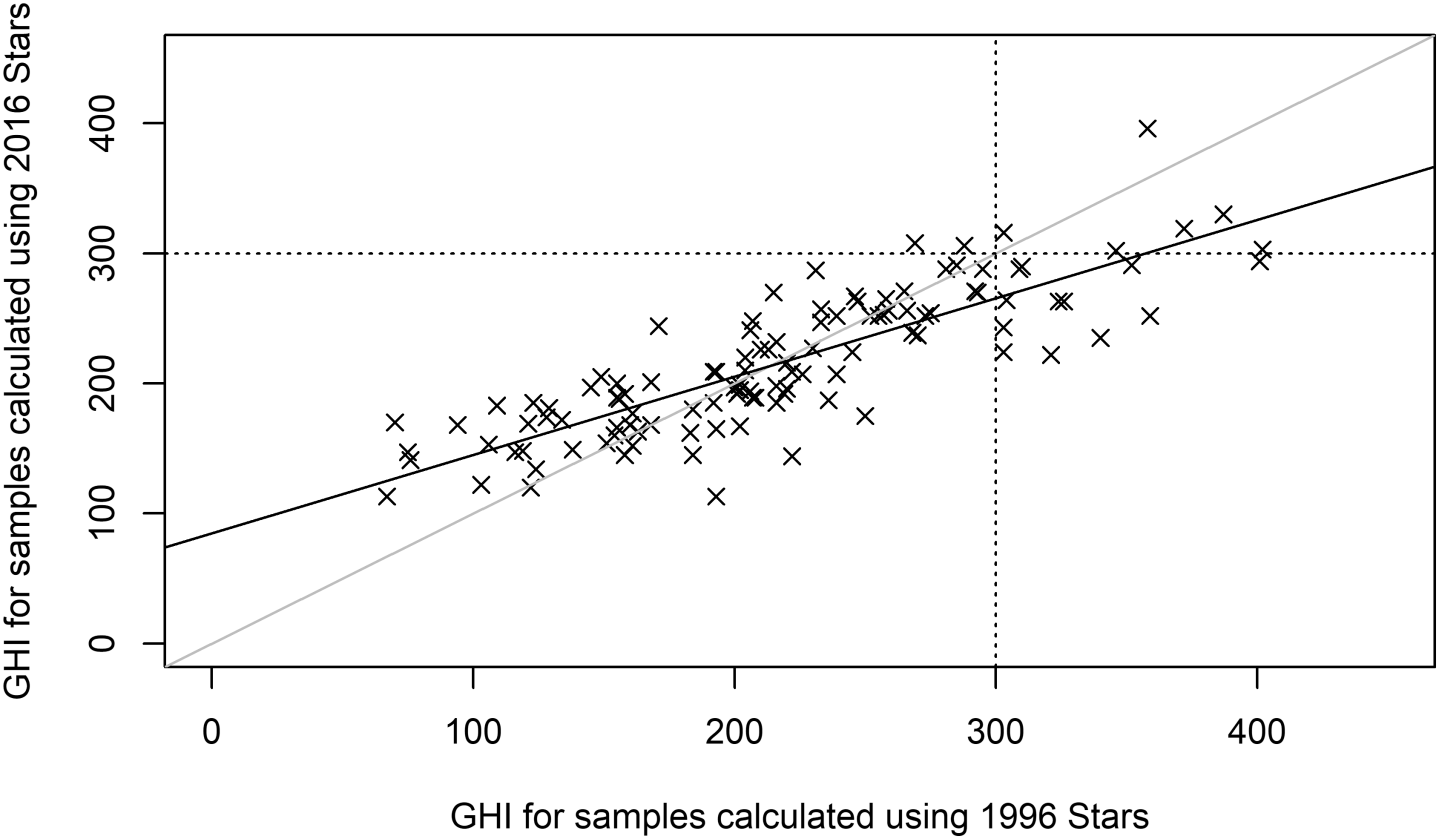
Correlation between GHIs calculated for 114 samples of southwest Ghana using the 2016 Stars, and the GHI of those same samples calculated using the 1996 Stars: *r*(112) = 0.84, *p* < .005. Gray diagonal line is the 1:1 line; black diagonal line is the best fit linear regression line; vertical and horizontal dashed lines show the samples with GHI >300 (“hotspots”) in 1996 and 2016, respectively.

There is strong positive correlation between the 1996 and the 2016 GHIs: *r*(112) = 0.84, *p* < .005 (Figure 3). A linear regression model was fitted to predict GHI scores in 2016 from GHI scores in 1996. A significant regression equation was found (*F*(1,112) = 260.45, *p* < .0005), with an adjusted *R* ^2^ of 0.697. GHI in 2016 is equal to 84.8 + 0.602*(GHI in 1996). Hotter samples have become cooler with time, while cooler samples have become hotter with time. This is the result of changes to the overall number of Blue and Gold Star species in particular: cooler samples, dominated by Green Star species in 1996, have received an uplift in GHI as 26% of original Green Star species were upgraded to Blue Star species in 2016. Hotter samples, with the highest concentrations of Black and Gold Star species, now have a reduced GHI as 83% of Gold Star species were downgraded (and were not replaced by new ratings, as Black Star species were).

In terms of conservation implications, we are concerned by both the magnitude and direction of change in the apparent conservation value of samples at the top end of the GHI scale, rather than the bottom. Using a score of 300 as a threshold for the hottest samples, of the 18 samples with scores above 300 in 1996, six samples remain above 300 in 2016. The remaining 10 samples now fall below 300, but still above 200, representing high conservation value. Only two samples fell below 300 in 1996 but now have scores >300. Scores at the top end of the GHI scale have generally decreased slightly with time, and thus, fewer samples are not protected when they later prove to warrant it.

### 3.2 Inferred genuine changes in conservation status

The locations of the 2015 samples were not identical to any previous sample locations, and so we have no data to describe changes to the species composition of individual samples over time. Excepting Ankasa, these are production (logging) reserves with accessibility dependent on which compartment is being logged at the time, and the fate of individual samples over time would in any case depend on whether or not it had just been logged or otherwise disturbed. Neither was the most recent survey effort equal to all previous survey efforts: Only 30 of the 114 samples considered were carried out in 2015. However, we did work in the same reserves, choosing to sample good condition samples of the apparent vegetation types in those same reserves (including in the GSBAs), using the same methods as in previous studies, so at the vegetation‐type level, the data collected in 2015 comprise an informative resurvey effort.

Two hundred and eighty‐six species were not refound in 2015, out of a total of 931 species recorded at any time from these reserves (30.7%). The 286 species which were not refound were not biased by 2016 Star (chi‐square test of association, X‐squared (df = 3, *n* = 931) = 1.91, *p* = .59). Nine Black Star species (*Monocyclanthus vignei*, *Pavetta abujuamii*, *Pavetta sonjae*, *Pavetta subglabra*, *Psychotria longituba*, *Psychotria nigrostellata*, *Schefflerodendron ekpei*, *Suregada ivorensis*, and *Synsepalum ntimii*) and 16 Gold Star species were not refound: These are potentially of concern, but given the lower sampling effort and the local rarity of these species we do not believe that they are truly missing. Eighty‐eight species are unique to the 2015 dataset, that is, recorded for the first time in the reserves in 2015, including two Black Star species (*Ficus pachyneura* and *Momordica sylvatica*) and four Gold Star species (9.45% of all species in the dataset). These 88 species are also not biased by Star (chi‐square test of association, X‐squared (df = 3, *n* = 931) = 0.55, *p*‐value = .91).

For all but the most widespread and well‐collected plants of west Africa's forest zone, changes to our perception of species' taxonomy and distributions strongly outweigh our ability to detect real distribution changes over time. The likelihood of genuine and significant range extensions over 20 years for these species can be dismissed as low, given the ecology of the species and habitat changes ongoing in the forest zone of west Africa (Stévart et al., 2019).

## 4 DISCUSSION

This study showed that there has been considerable flux in our conceptions of taxonomy and distributions for plant species within southwest Ghana over 20 years, an area that is well studied relative to other tropical endemism hotspots. 36% of species changed global AOO category (Star category), and 12% of species occurrences in our dataset have been contributed since 1996. Species tended to appear more widespread, rather than more restricted, over the 20‐year re‐evaluation period (20% vs 16%). This effect is most marked for the globally rarest species, with two thirds of local endemics moving to a more widespread AOO category after 20 years and just one third remaining in the same category. However, new species discoveries and new records for the region were disproportionately of globally rare species, so that the total number of local endemics known in southwest Ghana was maintained over the 20 years. This explains why mean endemism scores (bioquality scores) have remained constant over 20 years, even though the species responsible for the perception have changed. Importantly, new locally endemic species were found in the same broad locations as the now‐downgraded local endemics, in spite of random survey effort, so that the localities which appeared as hotspots of endemism in 1996 still appeared that way in 2016. This stability in assessment for localities allows for meaningful conservation priority setting over time and in the face of partial information.

This lability in documented ranges and conservation assessments is probably applicable to most groups of plants in the tropics. In neotropical *Myrcia*, for example, around 33% of species changed their apparent EOO‐based conservation status as the result of 10 years of specimen collection, databasing, and taxonomic changes (Nic Lughadha et al., 2019). Similar to our findings, transitions to smaller EOO categories outnumbered transitions to larger EOO categories (8% vs 3%, respectively), and species previously deemed data deficient transitioned to threatened status more often than to not threatened (10% vs 7%, respectively; Nic Lughadha et al., 2019). For Madagascan orchids, species described more recently have smaller ranges and occupancies, fewer specimens and greater perceived extinction risk status (Roberts et al., 2016). In the Cape Floristic Region, narrow‐range taxa have constituted a significantly greater proportion of species discoveries since 1950 (Treurnicht et al., 2017). The almost universal lability of taxonomic nomenclature has been perceived as awkward for conservation (Garnett & Christidis, 2017). Rather than indulge calls to “finalise” taxon names (Thomson et al., 2018), accepting a degree of lability in conservation assessments would be an alternative and more accurately reflect our evolving knowledge of the natural world.

Although some relatively common species were previously considered rare in our study, as a result of undersampling, no rare species were misclassified as common, so all the species that appeared worthy of attention at the time would have received it. Many rare species had been overlooked or were not yet described, and these were disproportionately found in original hotspot locations, despite equal resampling effort in cold and hot spots. The precautionary principle is often touted in conservation science (IUCN, 2016b), but it less often followed. When specimens represent the best available evidence for particular species, their use as a basis for extinction risk assessment is appropriate, necessary, and urgent (Lughadha et al., 2019). This case study supports the precautionary principle: The correct areas were given conservation funds despite undercollection limiting our knowledge of those plant species' distributions outside of the study area. Protected areas benefited from improved afforestation and rainfall patterns, reduced illegal tree felling and group hunting, less seasonal reduction of volumes of water bodies, and the cessation of the use of poisonous chemicals in fishing (GEF Evaluation Office–UNDP Evaluation Office, 2007).

We proffer that it is not our 250 years of plant biological records which are inappropriate for assessing plant conservation status, but rather the 22‐year‐old IUCN Red List system. Many authors have suggested approaches to accelerate Red Listing by offering more appropriate assessment alternatives for plants species. These focus particularly around using coarser distribution data while relaxing the requirement for or using crude proxies for decline in habitat area, extent or quality (Darrah et al., 2017; Le Breton et al., 2019; Marshall et al., 2016; Miller et al., 2012; Pelletier et al., 2018; Stévart et al., 2019). Another important nexus of work is to improve the online mobilization and dissemination of published botanic records from sources other than herbaria, including checklist data, Floras, and scientific publications, into data standards and repositories appropriate for conservation assessments (Agosti et al., 2022). As much as 39% of plant species selected randomly for inclusion in the Sampled Red List Index for Plants could not be evaluated against the Red List criteria, even as Data Deficient (Brummitt, Bachman, Griffiths‐Lee, et al., 2015). A conservation assessment system that cannot be applied to 39% of plant species, and likely the rarest and certainly the least well‐documented 39% at that, is surely too stringent. The problem with applying too stringent a set of criteria relative to the available evidence is that we fail to define a baseline against which to monitor change for many species. Rather than accept that many plant species cannot be assessed and continue to focus our efforts on only the most well‐known and perhaps least threatened plant species, we support calls to assess plant species using the evidence available and characterize how our current data may be biasing our perspective, but ultimately to accept some lability in those assessments.

We recognize that the goal of the Red List is to assess extinction risk in a comparable way across eukaryotes, rather than to assess a taxon's range (Collen et al., 2016). The relationship between the risk of extinction and global range (endemism) is not absolute, though narrow AOO or EOO is an all but essential prerequisite for a plant's assessment as threatened, and many restricted range species are threatened (Robbirt et al., 2006). Tropical plant species with narrow ranges declared extinct are least likely to be discovered extant (Humphreys et al., 2019). We suggest that species known from <5 specimens should not be considered as unassessable, or Data Deficient. The precautionary principle, which is already enshrined in the IUCN guidance, should be employed in practice. Such species could be assessed by their AOO, along with number of locations, and appropriate proxies or evidence for a decline in habitat area, extent or quality which could be relatively general in the case of plants. Our study suggests that such species may indeed prove to be more widespread (and less threatened) over time than they initially appeared. But in the meantime, their recognition and presence shines a light on an area which is poorly known and quite likely to harbor new records or species new to science, which has the added benefit of being afforded conservation protection, perhaps also helping to reduce the historical 70 years required to collect 15 geolocated specimen localities (Goodwin et al., 2020).

Herbaria are principally a taxonomic repository, retaining type specimens and significant range extensions such as new country records. Vouchered specimens remain essential to provide verifiable identifications for work in ecology, phylogenetics, taxonomy, conservation, and pharmacology (Funk et al., 2018). A new sort of herbarium specializing in, or at least accepting, recollections of species from known localities, specimens located within the known EOO, sterile vouchers, and specimens which describe the species of a locality in depth would be useful for conservation assessment and reassessment. Such specimens need not be curated to such a high specification as traditional specimens or even be kept at all, once photographed with a small amount of leaf material retained, for example, for DNA extraction. Specimens could be linked via QR codes or similar to online platforms such as iNaturalist, providing supplementary access to living plant photographs, identification history, location maps, and potentially additional geolocated records from the area, without increasing physical storage requirements (Heberling & Isaac, 2018).

We further suggest a greater emphasis is placed on area‐based conservation assessment than species‐level assessment (Plumptre et al., 2019). Habitat assessment is an extension from species‐level assessment, an approach adopted in the Star and bioquality system, as well as Key Biodiversity Areas and Tropical Important Plant Areas (TIPAs), for example, in Guinea (Couch et al., 2019). If all 22 Guinean TIPAs could be protected, over 60% of Guinea's threatened plant species and 100% of Guinea's Threatened Habitats would survive. Key Biodiversity Area assessment in Uganda showed that most of the remaining natural habitat was important for the conservation of globally and nationally threatened species and threatened habitat (Plumptre et al., 2019). Emphasizing area‐based assessments, such as range size rarity or weighted endemism, also reduces the importance of “fixing taxonomy” at the species level (Thomson et al., 2018). Our study shows that locality assessments, which make use of the summed (or averaged) signal from many species, were more stable over time that the assessment of any one individual species. By acting on the signal from the apparently rare species, some of which later turned out to more widespread, conservation priority areas were nevertheless identified and protected. It is surely not wise to imagine that we can ever finalize our estimates of AOO, EOO, Red List status, or nomenclature. Fortunately, the consequences of not doing so may be less severe than we imagine.

## AUTHOR CONTRIBUTIONS

**Cicely A. M. Marshall:** Conceptualization (lead); data curation (equal); formal analysis (lead); funding acquisition (equal); investigation (equal); methodology (equal); project administration (equal); resources (equal); writing – original draft (lead); writing – review and editing (lead). **Jonathan Dabo:** Data curation (equal); investigation (equal). **Markfred Mensah:** Data curation (equal); investigation (equal). **Patrick Ekpe:** Data curation (equal); investigation (equal); resources (equal). **William D. Hawthorne:** Data curation (lead); funding acquisition (equal); investigation (equal); methodology (equal); resources (equal); writing – review and editing (equal).

## CONFLICT OF INTEREST

The authors declare no competing interests.

## Data Availability

The data that support the findings of this study, including all plot data and species data, are publically available at Marshall, Cicely et al. (2023), Data for Implications for conservation assessment from flux in the botanical record over 20 years in southwest Ghana, Dryad, Dataset, https://doi.org/10.5061/dryad.qbzkh18n8.
